# The Relation between Resting State Connectivity and Creativity in Adolescents before and after Training

**DOI:** 10.1371/journal.pone.0105780

**Published:** 2014-09-04

**Authors:** Janna Cousijn, Kiki Zanolie, Robbert J. M. Munsters, Sietske W. Kleibeuker, Eveline A. Crone

**Affiliations:** 1 Brain and Development Lab, Department of Psychology, Leiden University, Leiden, The Netherlands; 2 Leiden Institute for Brain and Cognition, Leiden, The Netherlands; 3 Department of Developmental and Experimental Psychology, Utrecht University, Utrecht, The Netherlands; University of Granada, Spain

## Abstract

An important component of creativity is divergent thinking, which involves the ability to generate novel and useful problem solutions. In this study, we tested the relation between resting-state functional connectivity of brain areas activated during a divergent thinking task (i.e., supramarginal gyrus, middle temporal gyrus, medial frontal gyrus) and the effect of practice in 32 adolescents aged 15–16. Over a period of two weeks, an experimental group (n = 16) conducted an 8-session Alternative Uses Task (AUT) training and an active control group (n = 16) conducted an 8-session rule switching training. Resting-state functional connectivity was measured before (pre-test) and after (post-test) training. Across groups at pre-test, stronger connectivity between the middle temporal gyrus and bilateral postcentral gyrus was associated with better divergent thinking performance. The AUT-training, however, did not significantly change functional connectivity. Post hoc analyses showed that change in divergent thinking performance over time was predicted by connectivity between left supramarginal gyrus and right occipital cortex. These results provide evidence for a relation between divergent thinking and resting-state functional connectivity in a task-positive network, taking an important step towards understanding creative cognition and functional brain connectivity.

## Introduction

Adolescence is a time of natural exploration and learning, putting great demands on the ability to flexibly adapt to changing environments (e.g., learning new tasks in school, developing social competencies). The emergence and sophistication of the skills which support flexibility have been interpreted in terms of reorganization of brain networks which aid cognitive control [1], explorative learning [2] and social competence [3]. However, little is known about the relations between flexibility in finding solutions for day-to-day challenges that adolescents face when growing up, and connectivity between brain regions supporting this ability. We therefore examined the relation between flexibility in thinking and functional connectivity at rest, and how this network is sensitive to adaption due to training of flexibility in thinking in adolescents.

Creativity, which involves the ability to provide solutions that are novel and useful, is a key component of flexible thinking (for reviews see [4]–[6]). Creativity is often operationalized by asking participants to perform tasks relying on divergent thinking, such as the Alternative Uses Task (AUT) in which participants are asked to come up with as many possible alternative uses for daily objects [7]. Using an adapted version of the alternative uses task (AUT-scanner) Fink et al. [8], [9] showed involvement of the angular gyrus (AG) and supramarginal gyrus (SMG) during generation of alternative uses of everyday objects. Involvement of (predominately left) parietal-temporal areas like the AG extending into the middle temporal gyrus (MTG) and the SMG in verbal divergent thinking is a relatively consistent finding across studies with similar paradigms [4], [5]. In a recent study, we showed that adolescents and adults engaged the same neural network when thinking of alternative uses of common objects, involving the left AG and bilateral MTG, and left SMG [10]. In addition, the lateral prefrontal cortex (PFC) was more engaged depending on task performance. Adults were more successful in generating alternative uses compared to adolescents, which was reflected by higher levels of PFC activation in adults. These findings are consistent with other studies that pointed towards PFC involvement in divergent thinking, however, the location of activity within the PFC varied between studies (e.g., [11]–[14]).

Prior studies mainly examined the neural correlates of skills that adolescents employ when performing cognitive control tasks (for reviews see [15], [16]) but few studies have examined the adaptation of brain networks as an effect of practice or training. Studies that did examine changes in brain functionality as a result of training showed that especially prefrontal and parietal brain regions changed as a result of several days of math training [17] or five or six weeks of working memory training [18], [19]. Also, intensive inductive reasoning training altered patterns of functional connectivity between frontal and parietal cortex during rest [20]. Mackey et al. [20] asked Law School students to participate in a resting state (RS) connectivity study before and after a 3 month Law School Admission Training program. They showed that connectivity between brain regions involved in inductive reasoning, specifically frontal-parietal network areas, were dependent on experience such that they were modified by intensive training. Here we used this approach to examine experience dependent modification of connectivity during rest in adolescents using a creative cognition training paradigm.

The goal of the current study was twofold: investigate (1) the cross-sectional relation between divergent thinking performance and RS connectivity, and (2) the effects of a divergent thinking training on RS connectivity in adolescents. A prior study investigating divergent thinking and RS connectivity [21] found that divergent thinking was positively related to connectivity within the default mode network, that is to say, larger sum scores on a divergent thinking test (partly similar to the AUT) were related to stronger connectivity between the medial PFC and posterior cingulate cortex. However, this study did not examine connectivity in a task-related network (MTG/AG, SMG, and PFC). In addition, prior research suggested that improvement in task performance as a result of training could be associated with more brain plasticity in adolescence [17], but it is currently unknown how this relates to functional brain connectivity measures. We therefore tested the hypothesis that core regions involved in divergent thinking (MTG [extending into AG], SMG, and MeFG [Medial Frontal Gyrus] as part of the PFC) would show stronger connectivity at rest after a divergent thinking training, relative to a control group.

We randomly divided 32 adolescents into an experimental or active control group [19]. Over a period of two weeks, the experimental group conducted an 8-session AUT-training, whereas the active control group conducted an 8-session control training (rule switching), which was similar in time demands and effort but did not put demands on divergent thinking [19]. RS connectivity and measures of divergent thinking were assessed before (pre-test) and after (post-test) training. As previous research showed involvement of the MTG (including the AG), SMG, and PFC in divergent thinking, a seed based approach was used to specifically investigate RS connectivity of areas activated during a divergent thinking task in the same participants (i.e., left MTG including the AG, right MTG, left SMG, and bilateral MeFG). Second, an explorative whole-brain approach was used to more generally investigate RS connectivity within brain networks with a strong temporo-parietal or PFC component. We expected divergent thinking to be positively related to RS connectivity of the MTG, SMG, and MeFG at pre-test. Moreover, divergent thinking was expected to improve in adolescents following the AUT-training. In post hoc analyses we tested whether the behavioral training outcomes were related to RS connectivity of the MTG, SMG, and MeFG at the pre-test.

## Materials and Methods

### Participants

A total of 32 adolescents (18 male) aged 15 to 16 participated in this study (see Table 1 for sample characteristics). This age selection was based on a prior study that showed training effects in task-related activity in 15–16-year-old adolescents [17]. The participants were recruited from the general population through local advertisements. All participants were healthy, right-handed, and met MRI safety criteria (e.g., no braces or metal implants). None of the participants had a history of psychiatric or behavioral problems in the clinical range, which was assessed with the Child Behavior Checklist (CBCL [22]) and the Behavior Rating Inventory of Executive Function (BRIEF [23]). The participants were divided into an experimental group and a control group matched for gender. Groups did not significantly differ on IQ as estimated from the Wechsler Intelligence Scale for Children (WISC [24]) Similarities and Digit Span subtests (*t*_30_ = 1.16, *p* = 0.26). However, the experimental group was a few months younger than the control group (*t*_30_ = 2.40, *p* = 0.023). The study was approved by the Medical Ethics Committee of the Leiden University Medical Center (LUMC).

**Table 1:** Sample characteristics and cognitive assessments at pre-test and post-test.

	Experimental group		Control group^1^	
	pre-test	post-test	pre-test	post-test
N (males)	16 (9)	–	16 (9)	–
Age, mean (SD)	15.8 (0.1)	–	16.3 (0.1)*	–
IQ, mean (SD)	106.2 (11.5)	–	101.7 (10.6)	–
AUT-scanner fluency, mean (SD)	2.1 (0.6)	2.1 (0.7)	2.2 (0.4)	1.8 (0.4)^#^
AUT-brick fluency, mean (SD)	–	9.8 (3.2)	–	11.3 (5.2)
AUT-brick flexibility, mean (SD)	–	7.6 (2.0)	–	7.3 (2.3)
AUT-brick originality, mean (SD)	–	1.7 (0.4)	–	1.7 (0.3)
AUT-brick feasibility, mean (SD)	–	4.6 (0.4)	–	4.5 (0.4)
CAT fluency, mean (SD)	–	14.6 (3.6)	–	12.5 (2.6)
CAT originality, mean (SD)	–	27.7 (11.6)	–	22.1 (8.8)
MCT PC (SD)	0.89 (0.13)	0.93 (0.06)	0.88 (0.06)	0.90 (0.06)^ #^
LGT switch trials, PC (SD)	0.94 (0.07)	0.95 (0.08)	0.90 (0.08)	0.91 (0.07)
LGT switch trials, median RT (SD)	509.2 (129.1)	428.2 (69.3)	525.3 (211.31)	354.7 (47.2)**^##^

*p<0.05 and ** p<0.001 for group comparison.^ #^p<0.05 and ^##^p<0.001 for pre-test post-test main effect of time. AUT; Alternative Uses Test. CAT; Creative Ability Test. MCT; Mental Counters Task. LGT; Local Global Task. PC; Proportion Correct. RT; Reaction Time. SD; Standard Deviation. ^1^Brick data missing from one control participant.

### Cognitive assessments during pre-test and post-test

#### Alternative Uses Test-scanner (AUT-scanner)

Participants performed an adapted version of the Alternative Uses Test (AUT [25]) inside the MRI scanner (see also Kleibeuker, etal. [10]). This task measures divergent thinking in the verbal domain. The task consisted of an Alternative Uses (AU) condition and an Object Characteristics (OC) condition. During AU trials participants had to think of as many appropriate alternative and original uses of common objects as possible (e.g., use a shoe as a baseball bat). During OC trials participants had to think of as many ordinary characteristics of common objects as possible (e.g., a shoe fits on a foot). Each trial started with a 3 seconds instruction screen, followed by the target screen that lasted for 15 seconds. Immediately after the target screen, the participants had 3 seconds to indicate the number of generated solutions by pressing a response box button corresponding to ‘0–1′, ‘2′, ‘3′ or ‘4+’. Each trial was preceded by a fixation cross with a jittered duration (0–6 seconds). In the context of this study, the behavioral results of the AUT condition were used as an index of verbal divergent thinking. The AUT-scanner consisted of 60 trials divided over three blocks, during which 30 unique words were used (once presented in the AU and once in the OC condition). Two sets of 30 words were created, matched on word length, number of syllabi, and word frequency. A different word set was used during the pre-test and post-test and order of the two word sets was counterbalanced across participants. Each block lasted ∼7 minutes with a short break in between resulting in a total task time of ∼30 minutes. The task was programmed in E-Prime (version 2.0). AUT-scanner performance was measured by calculating the average number of solutions (fluency).

#### Alternative Uses Test-brick (AUT-brick)

The AUT-brick [26] was administered as a near transfer task during post-test only. Similarly to the AUT-scanner the AUT-brick measures divergent thinking in the verbal domain and performance on both tasks is strongly related [10]. Participants were instructed to generate as many appropriate alternative and original uses for a brick. Participants typed their solutions one at the time on a laptop during a period of 4 minutes. From the given solutions different measures were computed. *Fluency* scores were computed by counting the number of correct solutions provided. *Flexibility* scores were computed by counting the number of different solution-categories. An independent trained rater assigned each solution to one of 35 predefined solution-categories (e.g., building aspect, load, toy [27]). The number of different applied solution-categories was subsequently summed per participant. *Originality* was assessed on a 5-point scale (from 1 = “not original” to 5 = “highly original”). An independent trained rater rated each solution according to a developed rating scheme [27]. Originality scores were computed by averaging the ratings of all solutions per participant. *Feasibility* scores were computed by scoring the feasibility and usefulness of the generated solutions. An independent trained rater (TdeW) rated the feasibility of each solution. The feasibility scores of the generated solutions were subsequently averaged per participant.

#### Creative Ability Test (CAT)

The Creative Ability Test (CAT [28]) measured divergent thinking in the visuo-spatial domain and was administered as a far transfer task during post-test only. The participants viewed nine squares including dots. Per square, the dots could differ in location, color and number. Participants were instructed to find triads of squares based on the characteristics of the dots (i.e., number, color, location). *Fluency* scores were computed by counting the number of correct solutions provided. *Originality* scores were computed by summing the uniqueness score of each correct answer. Uniqueness (1 = “common” to 5 = “very unique”) was determined by the occurrence of a given solution in previous validation studies [29]. The time to complete the task was limited to 25 minutes.

#### Mental Counters Task (MCT)

Several studies reported a positive relationship between divergent thinking and working memory [30]. To examine training effects on working memory, working memory was assessed at pre-test and post-test with the Mental Counters Task (MCT; adapted from [31]). Participants were instructed to actively memorize the numerical value of independent counters. Following a blocked design, either two or three counters had to be simultaneously memorized. These counters were represented by horizontal lines, above or below which squares appeared. If the square appeared above the line ‘1′ had to be added to the value of the counter. If the square appeared below the line ‘1′ had to be subtracted from the value of the counter. At the start of a trial the participants were instructed to press a button when one of the counters reached a specific criterion value (e.g., ‘press the button when one of the counters reaches 3′). Each trial consisted of a series of 5 or 7 squares. A response was required within 3500 ms after the criterion was reached. Working memory scores were computed as the proportion correct trials.

#### Local Global Task (LGT)

The Local Global Task (LGT; adapted from [31]) measured rule-switching and was used as the active control task in the control training condition. During the task, large squares and rectangles (global figures) consisting of small squares or rectangles (local figures) were presented as target stimuli. Participants were instructed to indicate the shape (i.e., square or rectangle) of either the global or the local figure as indicated by the presence of a global or local cue. The global cue was a large square and rectangle, whereas the local cue was a small square and rectangle, presented on the left and right side of the target. Participants pressed a right or left response button corresponding to the correct (global or local) shape of the target. Each trial started with a 500 ms presentation of the global or local cue, after which the target stimulus would appear. Participants had 3500 ms to respond to the target stimulus (for details see [31]). The task consisted of a global and a local bock (each 50 trials, counterbalanced over participants), followed by a switch block (160 trials). The switch block consisted of alternating global and local mini blocks of 4 trials. From the switch block trials, median response times (RTs) and proportion correct of local-global switch trials were computed.

### Cognitive training

The experimental group performed an 8-session AUT-training at home, whereas the control group performed an 8-session Local-Global Task switching (LGT) training at home. Training length was based on a EEG study that demonstrated divergent thinking training effects in frontal brain activity in adults [32]. The AUT-training task resembled the AUT-scanner task; participants had to generate as many appropriate alternative and original uses of common objects as possible. Nine different objects were presented during each training session. The objects were different from the AUT-scanner objects. Word length, number of syllabi, and word frequency were matched across sessions. Participants were given 2 minutes to enter their solutions. *Fluency* scores were computed separately for each session. *Originality* and *feasibility* were computed as described above but only for the first and last session. After each AUT-training session the participants were asked to rate their motivation during the training on a 4-point likert scale ranging from ‘not at all’ to ‘very’. The LGT-training was similar to the LGT switch blocks at pre-test and post-test. Each training session consisted of 8 blocks of 40 trials with self-spaced breaks in-between blocks. Similar to the switch blocks of the LGT at pre-test and post-test, the LGT training blocks contained alternating global and local mini blocks of 4 trials. Median RTs and percentage correct of local-global switch trials were computed for each training session. Each training session lasted around 20 minutes.

### fMRI data acquisition and data pre-processing

http://www.gatesfoundation.orgA 3T MRI scanner (Philips Intera, Best, The Netherlands) at Leiden University Medical Center with a standard 32-channel whole-head coil was used for image acquisition. Participants were instructed to lie still with their eyes closed and not to fall asleep. Bold signal during rest was measured with a T2* gradient-echo EPI sequence (TR 2.2s, TE 30 ms, 38 slices, slice thickness 2.75 mm, FOV 220×220 mm, in-plane resolution 2.75×2.75 mm, flip angle 80°, sequential slice acquisition). A total of 140 volumes were acquired resulting in a scan time of 5 min. A high resolution T1 structural scan was acquired for anatomical reference (T1 turbo field echo, TR 9.8 ms, TE 4.6 ms, 140 slices, slice thickness 1.2 mm, FOV 224×178 mm, in-plane resolution 0.88×0.88 mm, flip angle 8°). Data pre-processing was conducted with FEAT (FMRI Expert Analysis Tool) version 6.0, part of FSL (FMRIB’s Software Library, www.fmrib.ox.ac.uk/fsl). Preprocessing consisted of removing non-brain tissue with BET [33], slice-time correction, motion correction using MCFLIRT [34], high-pass filtering in the temporal domain (sigma = 100s), spatial smoothing with a 4 mm Gaussian kernel, and prewhitening [35]. Functional data were registered to the participants’ structural image and transformed to MNI space (Montreal Neurological Institute). Absolute motion from the middle volume to any other volume did not exceed 0.65 mm in any of the RS-fMRI scans. Relative motion from one volume to the next volume did not exceed 0.17 mm in any of the RS-fMRI scans. Moreover, motion parameters did not significantly differ between groups or showed a group x time interaction (*p*>0.33).

Bold signal during the AUT-scanner was measured with a T2* gradient-echo EPI sequence (TR 2.2s, TE 30 ms, 38 slices, slice thickness 2.75 mm, FOV 220×220 mm, in-plane resolution 2.75×2.75 mm, flip angle 80°, sequential slice acquisition). Each of the 3 task blocks represented a separate 7-minute run during which 167 volumes were acquired. Brain activity during the AUT-scanner was analyzed using SPM8 (Welcome Trust Centre for Neuroimaging, London, UK).

Images were first slice-time corrected and motion corrected. Inspection of the individual movement parameters ensures that none of the participants’ motion exceeded 3 mm in any direction. The functional images were subsequently normalized to the MNI305 stereotaxic space template (implemented in SPM8) using a 12-parameter affine transformation together with a nonlinear transformation involving cosine basis functions. Moreover, the data were resampled to 3×3×3 mm voxels and smoothed using an 8 mm full-width half-maximum isotropic Gaussian kernel.

### Procedure

Before the start of the first test-session, the participant and accompanying primary caregiver received general study information and had the opportunity to ask questions. All participants as well as their primary caregiver signed informed consent before participation. First, a training schedule was made together with the participants and their primary caregivers to facilitate training completion. The participants then briefly practiced the AUT-scanner task outside the scanner. This practice was immediately followed by the scanning session during which RS-fMRI data were acquired first, followed by the AUT-scanner and T1 structural scan. The accompanying primary caregiver filled out the CBCL and BRIEF during the scanning session. The WISC Similarities and Digit Span subtests were administered after the scanning session. Finally, the MCT and LGT (order counterbalanced over participants) were administered. Participants received 20 Euros compensation for completing the pre-test.

Training session progress was tracked online by the experimenter. In case a training session was forgotten, a text-message was sent to the participant’s cell phone to suggest another date to catch up. If a second training session was missed, the participant was called to discuss a new schedule.

The post-test session was planned exactly two weeks after the pre-test session. The participants were briefly informed about the procedure of the post-test and all participants as well as their primary caregiver signed informed consent before the post-test started. Similarly as at pre-test, RS-fMRI data were acquired first, followed by the AUT-scanner task and T1 structural scan. After the scanning session participants first performed the AUT-brick, CAT, followed by the MCT and LGT (order counterbalanced over participants). Finally, participants received 80 Euros for completing the training and post-test.

### Behavioral analysis

Behavioral scores from pre-test and post-test (AUT-scanner, MCT, LGT) were compared using repeated measures analyses of variance (rANOVAs) with group as between subject factor and time (pre-test, post-test) as within subject factor. AUT-brick data was missing for one control participant. Behavioral scores from the AUT-brick and CAT at post-test were compared between groups with standard t-tests. Four participants missed one AUT-training, two participants missed one LGT-training, and motivational ratings were incomplete for four participants. Moreover, given the current design, the covariance between sessions may differ (violation the assumption of sphericity). Behavioral scores during the AUT and LGT training were therefore compared within groups over time following a linear mixed-model approach [36]. Post-hoc t-test were Bonferroni corrected for multiple comparisons. Analyses were conducted with SPSS for Windows (IBM Corp. Released 2011. IBM SPSS Statistics for Windows, Version 20.0. Armonk, NY: IBM Corp.).

### fMRI analyses AUT-scanner at pre-test

**Figure 1:**
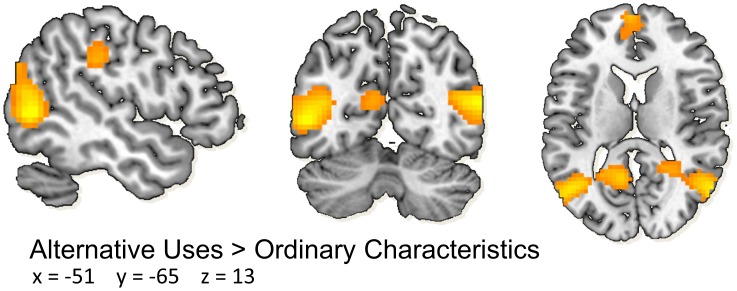
Brain activity for AU>OC at pretest. AU>OC at pretest resulted in activation in bilateral middle temporal gyrus, medial frontal gyrus and left supramarinal gyrus. Significant activations were observed in the bilateral middle temporal gyrus, medial frontal gyrus and left supramarinal gyrus. Significant clusters (>10 continues voxels, *p*<0.05 FWE-corrected) are overlaid on a standard MNI brain. Right side of the brain is depicted at right side. AU; alternative uses. OC; ordinary characteristics.

Statistical analyses were first performed on the individual level using the general linear model (GLM) in SPM8. For each participant, the functional time series were modeled with a series of events convolved with a canonical hemodynamic response function (HRF). Similarly to Kleibeuker et al. [10], trials were modeled separately for the AU and OC condition, with the instruction (0 duration), trial duration (15 seconds) and response window (0 duration) entered in the GLM as the events of interest, along with a basic set of cosine functions to high-pass filter the data, and a covariate for run effects. Individual contrast images were created for the AU>OC trial duration contrast using the least-squares parameter estimates of height of best-fitting canonical HRF. We used these contrast images in a group analysis to compute the main effect of AU>OC at pre-test (see Figure 1). Activity was considered significant if it consisted of at least 10 continuous voxels, *p*<0.05 FWE-corrected for multiple comparisons. This analysis served to create seed regions for the RS analyses.

### RS analyses

The RS analyses were performed in two ways. First, to investigate functional connectivity of brain areas involved in divergent thinking, a seed-based RS fMRI analysis was conducted. Second, to further explore the relationship between divergent thinking and RS connectivity in frontal-parietal brain networks, a RS-fMRI analysis was carried out using a standard group probabilistic ICA analysis (PICA [37]). Details on each of these analyses are described in the following sections.

#### Seed-based analysis

The AG extending into the MTG, SMG, and the various areas within the PFC have repeatedly been found activated during verbal divergent thinking tasks [4], [8]–[10]. Location of the seeds was therefor determined by the peak activity within these regions during performance of the AUT-scanner for the main effect of the AU>OC contrast across groups at pre-test (see Figure 1 and result section 3.2.1).

**Figure 2:**
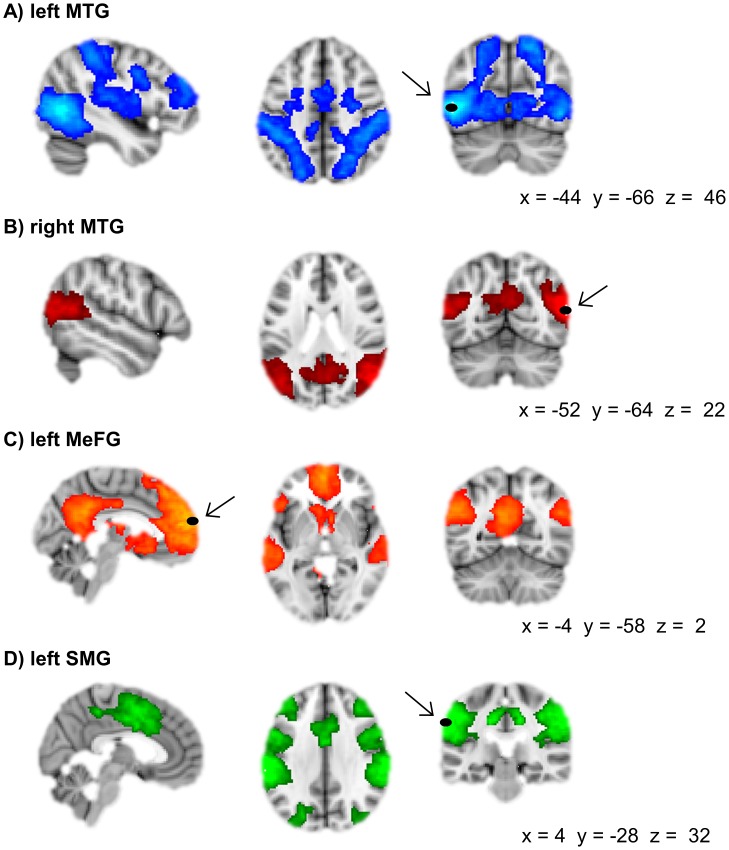
Seed regions of the seed-based resting-state analysis. Seed regions and their pattern of functional connectivity during rest at pre-test across groups are depicted. Seeds were based on fMRI data collected in the same participants while performing the AUT task at pre-test (see supplement) (**A**) Seed in the left middle temporal gyrus (left MTG), significantly connected to the right middle temporal gyrus and the bilateral supramarginal gyrus, precentral gyrus, occipital cortex, superior parietal cortex, and superior frontal gyrus. (**B**) Seed in the right middle temporal gyrus (right MTG) significantly connected to the left middle temporal gyrus and bilateral occipital cortex and precuneus. (**C**) Seed in the left medial frontal gyrus (MeFG) significantly connected to the right medial frontal gyrus and the bilateral precuneus, posterior cingulate cortex, inferior parietal cortex, middle temporal gyrus, caudate nucleus, and anterior cingulate cortex. (**D**) Seed in the left supramarginal gyrus (left SMG) significantly connected to the right supramarginal gyrus and bilateral superior parietal cortex, cingulate gyrus, precentral gyrus, middle frontal gyrus, and insula. A 4 mm sphere was drawn around the 4 peak voxels in the MTG, SFG, and SMG for the main effect of AU>OC across the experimental and control group. Clusters of significant functional connectivity (*Z* >2.3, whole-brain cluster corrected at *p*<0.05) are overlaid on a standard MNI template. Right side of the brain is depicted at the right side.

Binary masks were created for each seed voxel with a 4 mm sphere around it with the fslmaths function implemented in FSL (Figure 2). Seed masks were registered to the individual subject space and participant’s average signal time course was extracted from each seed region. The individual time courses from the seed region together with 9 nuisance parameters (white matter signal, CSF signal, global signal, six motion parameters; [38]) were entered as regressors in a General Linear Model (GLM) implemented in FEAT. GLM analyses were run separately for each participant and each seed region with the preprocessed RS-fMRI data in MNI space as input. All higher-level regression analyses and group analyses of the resulting contrast images were conducted using FLAME (FMRIB’s local analysis of mixed effects) stages 1 and 2. First, the linear relationship between AUT-scanner fluency and functional connectivity at pre-test was investigated. Second, connectivity patterns were compared between the experimental and control group over time. Finally, it was investigated if functional connectivity at pre-test predicted changes in AUT-scanner fluency over time (i.e., fluency post-test - fluency pre-test). Differences in connectivity were considered significant if *Z*>2.3, with a whole-brain cluster correction [39] of *p*<0.0125 (Bonferroni corrected for the number of seed areas). Significant clusters were localized with the Talairach Daemon database implemented in FSL and the LONI probability atlas [40].

#### Whole-brain analysis

PICA [37] is implemented in MELODIC (Multivariate Exploratory Linear Decomposition into Independent Components) version 3.13, part of FSL. The normalized RS-fMRI data were first resampled to 4-mm voxels to reduce computational burden. PICA preprocessing steps further included removal of non-brain voxels, voxel-wise de-meaning of the data, and normalization of the voxel-wise variance. Subsequently, a single 4D dataset was created by concatenating all RS-fMRI data in time. The concatenated data was projected into a 30-dimentional subspace using Principal Component Analyses (PCA) and decomposed into 30 spatially Independent Components (ICs) plus Gaussian noise using the FastICA algorithm [41], which optimizes the non-Gaussian spatial source distribution. ICs reflect both signal and artifacts in the data, consisted in space with internally consistent temporal dynamics across all participants and sessions. The resulting IC maps were divided by the standard deviation of the residual noise and thresholded with a 0.5 posterior probability threshold [37]. Each IC is finally represented by a time course and a spatial map of normalized Z-scores reflecting the degree to which a given voxel is significantly modulated by that time course. Given the suggested role of temporo-parietal areas and the PFC in divergent thinking, the analysis focused on brain networks with a strong temporo-parietal and/or prefrontal component. After PICA decomposition, the ICs with a strong temporo-parietal and/or prefrontal component were visually identified. ICs with motion artifacts, scanner artifacts, cardiac artifacts, and a mean power above 0.1 Hz were excluded.

Higher-level group analyses of connectivity within these networks were conducted using a validated dual regression approach [42]. First, individual connectivity maps were reconstructed from each group network map by using the network time-courses in a multiple linear regression against the individual RS-fMRI data in standard space resolution (2 mm). Second, statistical analysis of the individual connectivity maps was conducted using non-parametric permutation testing with 5000 permutations. To specifically investigate connectivity *within* the spatial boundaries of each network and to reduce the number of comparisons, the analyses were masked with the network’s spatial map as identified with the PICA analysis. The resulting t-maps were thresholded with a TFCE (Threshold-Free Cluster Enhancement) approach [43], *p*<.05 corrected for multiple comparisons. Similarly to the seed-based analysis, the linear relationship between AUT-scanner fluency and functional connectivity at pre-test was investigated. Moreover, connectivity patterns were compared between groups over time. Finally, it was investigated if functional connectivity at pre-test could predict changes in AUT-scanner fluency over time.

## Results

### Behavior

#### AUT-training

**Figure 3:**
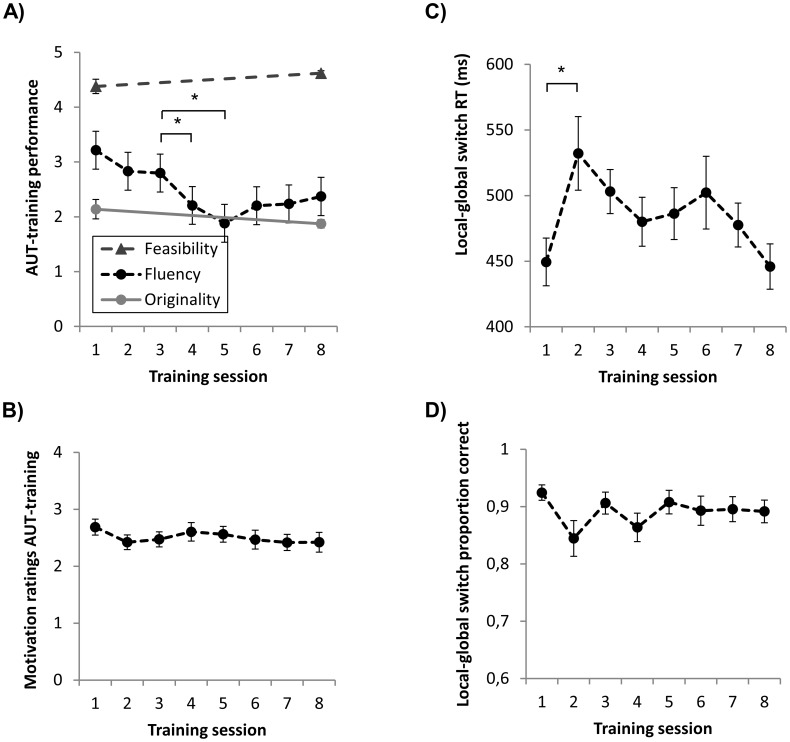
Behavior scores during the AUT-training and LGT-training. (**A**) Mean (standard error) feasibility, fluency, and originality scores of each AUT-training session in the experimental group (n = 16). (**B**) Mean (standard error) motivation scores of each training session in the experimental group (n = 16). (**C**) Median (standard error) reaction time of local-global switch trials of each LGT-training session in the control group (n = 16). (**D**) Mean (standard error) proportion correct of local-global switch trials of each LGT-training session in the control group (n = 16). * *p*<0.05, ** *p*<0.001.

Linear mixed-model analysis indicated that fluency scores significantly changed over the 8 training sessions (*F*_7,93.8_ = 3.31, *p* = 0.003, *Ω = *0.09). Fluency significantly dropped from session 3 to session 4 (*p*_corr_ = 0.045, *d = 0.81, 95% CI [0.01,1.18])* and 5 (*p*_corr_ = 0.009, *d = 1.70, 95% CI [0.13,1.71]*), see Figure 3A. However, originality (*t*_13_ = 1.30, *p = *0.22, *d = 0.52, 95% CI [−0.20,0.73]*) and feasibility (*t*_13_ = 1.90, *p = *0.08, *d = 0.50, 95% CI [−0.47,0.05]*) scores did not significantly change between training session 1 and 8 (Figure 3A). These results remain similar when correcting for motivational scores. Moreover, linear mixed-model analysis indicated that motivation did not significantly change over the 8 training sessions (*F*_7,82.9_ = 0.88, *p = *0.53, Figure 3B).

#### LGT-training

Linear mixed-model analysis indicated a main effect of time on median RTs of the local-global switch trials (*F*_7,85.2_ = 3.64, *p = *0.002, *Ω = *0.07). RTs significantly increased from session 1 to session 2 only (*p*_corr_ = 0.001, *d = *1.21, 95% CI [−154.9, −23], Figure 3C). Regarding proportion correct, linear mixed-model analysis indicated that the number of errors did not significantly change over the 8 training sessions (*F*_7,78.8_ = 2.00, *p = *0.066, Figure 3D).

#### Pre-test and post-test group analyses

Regarding the AUT-scanner, there was a main effect of time (*F*_1,30_ = 6.68, *p = *0.015, *η* ^2^ = 0.18) and an interaction between time and group (*F*_1,30_ = 4.25, *p = *0.048, *η* ^2^ = 0.12) on fluency scores. Such that, fluency scores remained stable in the experimental group (*p_corr_* = 0.71) but significantly decreased in the control group (*p_corr_* = 0.003, *d = *0.91, 95% CI [0.16,0.69], Table 1).

Regarding the LGT, there was a main effect of time (*F*_1,30_ = 23.2, *p*<0.001, *η* ^2^ = 0.44) but no significant interaction between time and group (*F*_1,30_ = 2.93, *p = *0.097) on local-global switch trial RT. Median RTs decreased in the experimental (*p_corr_* = 0.036, *d = *2.04, 95% CI [5.57,156.56], Table 1) and control group (*p_corr_* <0.001, *d = *1.28, 95% CI [95.10,246.09]). An analysis focusing only on post-test trials showed that at post-test, the control group was significantly faster than the experimental group on local-global switch trials (*p_corr_* = 0.001, *d = *1.42, 95% CI [30.70,116.30], Table 1). There were no significant effects of time (*F*_1,30_ = 0.21, *p = *0.65) or interaction between time and group (*F*_1,30_ = 0.06, *p = *0.80) on proportion correct, indicating that the number of errors remained stable over time regardless of training.

Regarding the MCT testing working memory, there was a main effect of time (*F*_1,30_ = 4.74, *p = *0.037, *η* ^2^ = 0.14) but no interaction between time and group (*F*_1,30_ = 0.21, *p* = 0.65). MCT performance increased across groups (*p_corr_* = 0.037, *d = *0.41, 95% CI [−0.06,0.00]), with no difference between groups (*p_corr_* = 0.34), indicating that working memory improved over time regardless of training.

Finally, regarding the near transfer and far transfer tasks; there was no significant difference between groups on AUT-brick fluency (*t*_29_ = 1.02, *p = *0.32), AUT-brick flexibility (*t*_29_ = 0.39, *p = *0.70), AUT-brick originality (*t*_29_ = 0.04, *p = *0.97), and AUT-brick feasibility (*t*_29_ = 0.52, *p = *0.60). Similarly, fluency (*t*_29_ = 1.91, *p = *0.07) and originality (*t*_29_ = 1.53, *p = *0.14) scores of the CAT did not significantly differ between groups (Table 1).

All analyses described above were run a second time with age as an additional covariate. Results and interpretations remain similar.

### Seed-based RS analysis

#### Seed locations

The main effect of the AU>OC contrast across groups during performance of the AUT-scanner at pre-test (Figure 1) revealed four peaks within the MTG, SMG, and PFC that were subsequently used as seed areas: left MTG: (x = −51, y = −66, z = 3, Z_max_ = 5.72), right MTG (including the AG; x = 57, y = −66, z = 15, Z_max_ = 5.89), left MeFG as part of the PFC (x = −3, y = 63, z = 18, Z_max_ = 4.84), and left SMG (x = −63, y = −27, z = 36, Z_max_ = 5.22]. The Main effect AU>OC during the alternative uses task in the scanner (AUT-scanner) at pre-test (n = 32) provided a replication of earlier findings [10].

#### Functional connectivity of the seed areas at pre-test across groups

Figure 2 shows the connectivity patterns of each of the seed areas across the experimental group and control group at pre-test. The left MTG showed significant connectivity to the right MTG and the bilateral SMG, precentral gyrus, occipital cortex, superior parietal cortex, and superior frontal gyrus. The right MTG showed significant connectivity to the left MTG and bilateral occipital cortex and precuneus. The left MeFG showed significant connectivity to the right MeFG and the bilateral precuneus, posterior cingulate cortex, inferior parietal cortex, MTG, caudate nucleus, and anterior cingulate cortex. Finally, the left SMG showed significant connectivity to the right SMG and bilateral superior parietal cortex, cingulate gyrus, precentral gyrus, middle frontal gyrus, and insula.

Table 2 gives an overview of the seed-based resting state analyses results, which are described in more detail in the following sections.

**Table 2:** Results of the seed based RS analyses.

seed area		analysis	significant area	side	direction	size (voxels)	MNI coordinates			Z_max_
							X	y	z	
***left MTG***										
*pre-test connectivity*										
	pre-test AUT-scanner fluency		ns.							
	pre-test post-test change AUT-scanner fluency		ns.							
*pre-test to post-test connectivity*										
	experimental group		SMG	R	positive	224	46	−40	40	3.12
	control group		inferior frontal gyrus	L	positive	217	−44	42	10	3.58
	group difference		ns.							
***right MTG***										
*pre-test connectivity*										
	pre-test AUT-scanner fluency		postcentral gyrus	L	positive	814	−34	−34	58	3.73
			postcentral gyrus	R	positive	650	32	−26	54	2.25
	pre-test post-test change AUT-scanner fluency		ns.							
*pre-test to post-test connectivity*										
	experimental group		ns.							
	control group		superior frontal gyrus	L	negative	233	−16	36	50	3.7
	group difference		ns.							
***left MeFG***										
*pre-test connectivity*										
	pre-test AUT-scanner fluency		ns.							
	pre-test post-test change AUT-scanner fluency		ns.							
*pre-test to post-test connectivity*										
	experimental group		ns.							
	control group		SMG	L	negative	246	−62	−42	38	3.70
	group difference		ns.							
***left SMG***										
*pre-test connectivity*										
	pre-test AUT-scanner fluency		ns.							
	pre-test post-test change AUT-scanner fluency		occiptital cortex	R	negative	941	30	−62	−16	5.01
*pre-test to post-test connectivity*										
	experimental group		Precuneus	L/R	positive	469	−4	−64	42	3.89
			SMG	R		426	40	−64	44	3.55
	control group		ns.							
	group difference		ns.							

MNI coordinates and Z-scores are shown of local maxima for each significant cluster. Statistical voxel threshold Z >2.3, critical cluster p = 0.0125 (corrected for the number of seeds). L: left. R: right. MNI: Montreal Neurological Institute. Ns.: non-significant. MTG: middle temporal gyrus. MeFG: medial frontal gyrus. SMG: supramarginal gyrus.

#### Relation pre-test RS connectivity and pre-test AUT performance across groups

**Figure 4:**
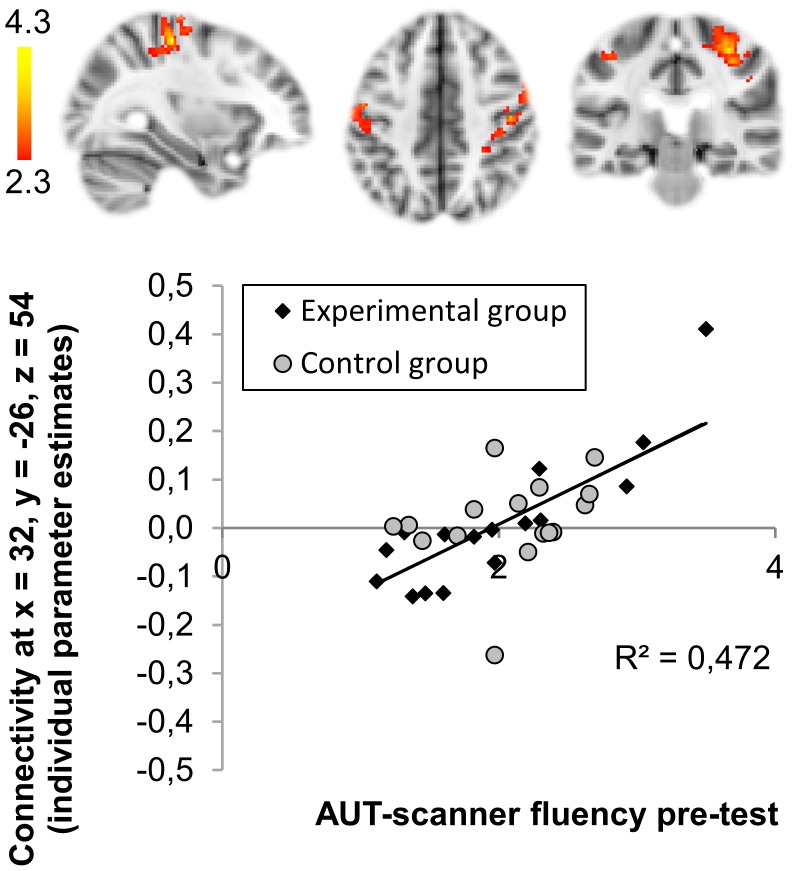
Relationship AUT-scanner fluency at-pre-test and right middle temporal gyrus connectivity to bilateral postcentral gyrus at pre-test. Higher fluency scores at pre-test are related to stronger functional connectivity at pre-test. Scatterplot depicts relationship between fluency and individual parameter estimated of connectivity at maximum (x = 32, y = −26, z = 54) separately for the Experimental (black) and Control (gray) group. Regression line depicts this association across groups. Clusters of significant functional connectivity *(Z* >2.3, whole-brain cluster corrected at *p*<0.0125) are overlaid on a standard MNI template. Right side of the brain is depicted at the right side.

AUT-scanner fluency at-pre-test was significantly positively related with right MTG connectivity to the bilateral postcentral gyrus (Figure 4), indicating that higher fluency scores were related to stronger connectivity between the right MTG and the postcentral gyrus. Across groups at pre-test, the right MTG and postcentral gyrus were not significantly connected (Figure 2) suggesting that this connectivity is present only for well performing participants. No other significant relations were observed between seed-based connectivity and AUT-scanner fluency at pre-test.

#### Relation training and RS connectivity over time

Comparing seed-based connectivity patterns between groups over time did not reveal any significant main or interaction effects, indicating that seed-based connectivity did not significantly change over time across groups and did not differ as a function of training. For descriptive purposes, we also compared seed-based connectivity patterns for each group separately, these findings are presented in Table 2.

#### Relation pre-test RS connectivity and change in AUT performance over time

Across groups, left SMG connectivity to right occipital cortex was significantly negatively related to change in AUT-scanner fluency scores over time. That is, a stronger anti-correlation between left SMG and right occipital cortex activity was associated with a larger increase in fluency.

### Whole-brain RS analysis

#### Network selection

**Figure 5:**
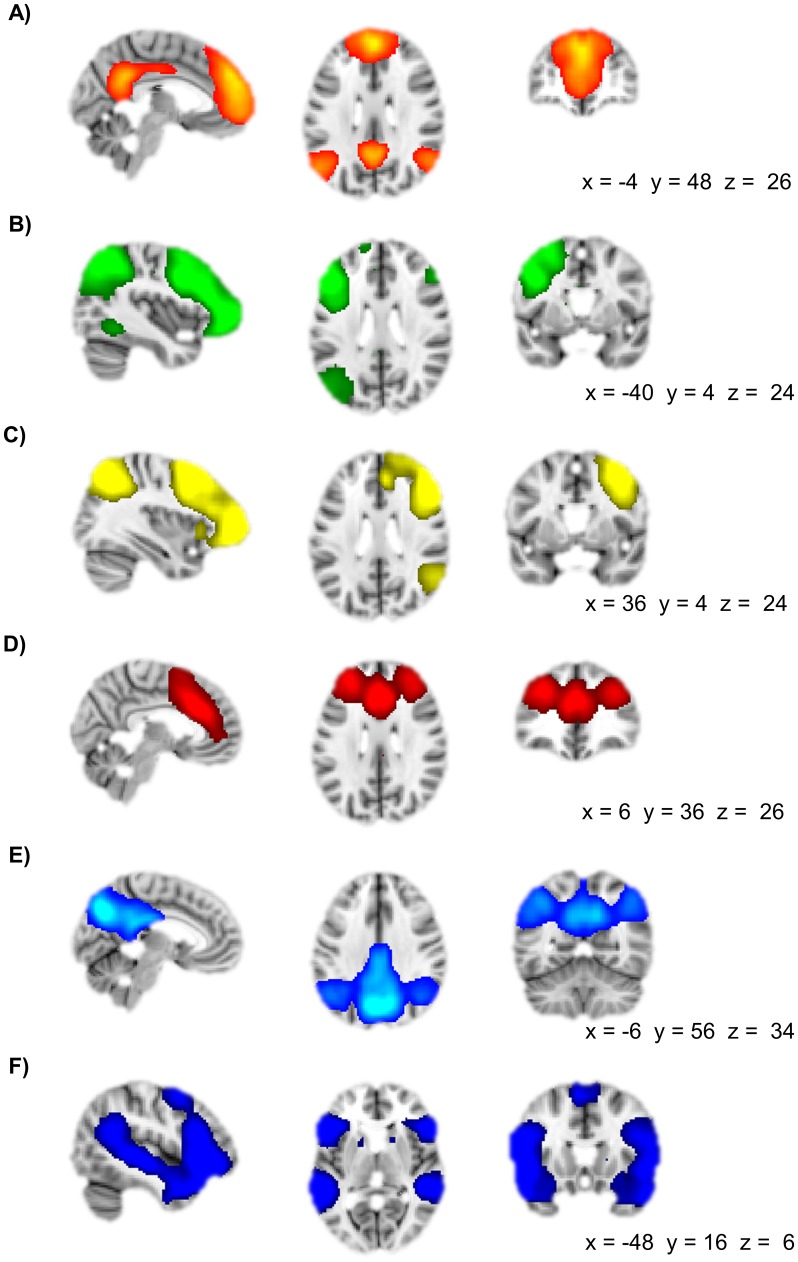
Spatial characteristics of brain networks included in the whole-brain RS analysis. (**A**) Default mode network including precuneus, posterior cingulate cortex, inferior parietal cortex, medial frontal gyrus, and anterior cingulate cortex. (**B**) Left frontal-parietal network including middle and superior frontal gyrus, superior parietal cortex, and middle temporal gyrus. (**C**) Right frontal-parietal including middle and superior frontal gyrus, superior parietal cortex, and meddle temporal gyrus. (**D**) Cognitive control network including middle and superior frontal gyrus, and dorsal part of anterior cingulate cortex. (**E**) Visuo-spatial attention network including precuneus, posterior parietal cortex, and posterior cingulated cortex. (**F**) Ventral visual stream including superior temporal gyrus and superior frontal gyrus. Significant clusters are overlaid on a standard MNI brain. Right side of the brain is depicted at right side.

Six literature consistent [44]–[46] RS networks were identified with strong involvement of temporo-parietal and/or prefrontal areas: default mode (precuneus, posterior cingulate cortex, inferior parietal cortex, MeFG, anterior cingulate cortex), left and right frontal-parietal (middle and superior frontal gyrus, superior parietal cortex, MTG), cognitive control (middle and superior frontal gyrus, dorsal part of anterior cingulate cortex), visuo-spatial attention (precuneus, posterior parietal cortex, posterior cingulated cortex), and ventral visual stream (superior temporal gyrus, superior frontal gyrus), see Figure 5.

#### Relation RS connectivity and AUT performance before and after training

Comparing connectivity patterns within the six RS networks between groups over time and within group over time did not reveal any significant main or interaction effects, indicating that connectivity patterns within these six RS networks did not significantly change as a function of training. AUT-scanner fluency at pre-test was not significantly related to pre-test functional connectivity in any of the six RS networks. Moreover, pre-test functional connectivity in any of the six RS networks did not significantly predict changes in AUT-scanner fluency over time.

## Discussion

The goal of this study was to investigate the relation between RS connectivity and verbal divergent thinking in adolescence before and after training. To this end, divergent thinking scores were related to functional RS connectivity patterns in task-relevant brain regions (bilateral MTG and MeFG, and left SMG) before and after divergent thinking training using a seed-based analyses approach. Moreover, a whole-brain ICA RS analyses was performed to further explore the relationship between divergent thinking and RS connectivity in brain networks with a strong frontal-parietal component. The study resulted in several important findings; (1) stronger connectivity between MTG and bilateral postcentral gyrus was associated with better divergent thinking performance, (2) training did not improve divergent thinking or change RS functional connectivity in the training group, and (3) even though there was no main effect of training on connectivity, the change in divergent thinking performance over time was predicted by connectivity between left SMG and right occipital cortex. The discussion is organized along the lines of these three main findings.

### Connectivity patterns and relation with performance at pre-test

The first question which was addressed was whether brain regions which have previously been implicated in divergent thinking [8]–[10] showed connectivity with each other at rest. For this purpose, we specifically looked at functional connectivity patterns at rest of brain areas activated during the performance of the AUT-scanner in the same participants: the MTG (including the AG), MeFG, and SMG (see Figure 1 and [10]). As predicted, the time course of activity within these regions showed significant correlations with each other, across hemispheres (see also [20]). These patterns of functional connectivity were further confirmed in a whole-brain resting state analysis, which mirrored previously reported RS networks [44]–[46].

A test for relations between functional connectivity patterns and performance revealed that higher AUT-scanner fluency scores were related to stronger connectivity between the right MTG and the postcentral gyrus. In addition to the seed-based analysis, the whole brain RS ICA analyses did not reveal any additional association between AUT-scanner performance and functional connectivity outside the seed areas. The connection between MTG, a region previously implicated in divergent thinking [8]–[10], and postcentral gyrus may aid in simulating possible ways in which objects can be used across cognitive and somatosensory domains, and as such may be helpful in thinking of more alternative uses. A prior study which investigated divergent thinking and RS connectivity already showed that divergent thinking performance moderated connectivity patterns in adults [21], however, this study specifically investigated functional connectivity of the default mode network, not the MTG. Takeuchi et al. [21] found that higher divergent thinking scores were related to stronger connectivity between the MeFG and posterior cingulate cortex. Here, we were able to make use of task-relevant seed areas derived from the same participants when performing the AUT while fMRI data were collected. Extending the prior findings of Takeuchi et al. [21], divergent thinking performance is also associated with stronger connectivity within a task-relevant network, specifically between MTG and the postcentral gyrus.

An important issue to take into consideration is that the generated alternative uses during the AUT-scanner were not recorded due to methodological difficulty. Hence, only a self-reported fluency estimate was available as a measure of AUT-scanner behavioral performance, not a measure of flexibility, feasibility and originality. Moreover, the observed relation between pre-test RS connectivity of the right MTG and AUT-scanner fluency at pre-test relies on the assumption that the participants correctly and honestly indicated the number of generated alternative uses. Similarly as reported by Kleibeuker et al. [10], a post-hoc analysis indicated that AUT-scanner fluency correlated with AUT-brick fluency (*r* = 0.39, *p* = 0.03) and flexibility *(r* = 0.49, *p* = 0.005), increasing the confidence in the AUT-scanner as a measure of verbal divergent thinking. Nevertheless, we cannot exclude potential confounding effects of the self-report measure of the number of generated alternative uses in the scanner. Future studies should replicate these findings using verbal creativity measures which can be scored on different creativity dimensions (e.g., flexibility, originality).

### Evaluating training outcomes behaviorally

The second question we addressed was whether a two week training program of divergent thinking would be sufficient to change divergent thinking performance in adolescents, similar to what previously has been shown for adults [32]. The pre-test to post-test comparison of fluency scores in the scanner resulted in the expected training group x time interaction, however, the findings were in a different direction than anticipated. That is, the experimental group outperformed the active control group at the post test, but this was due to a decrease in performance in the active control group at post-test compared to pre-test. A closer inspection of the training data showed that when evaluating performance across the home training sessions, the AUT-training did not improve in divergent thinking fluency scores. Thus, consistent with the AUT-scanner performance, AUT-training performance stayed the same across sessions, with a slight dip in fluency performance during session four and five. Notably, the active control group outperformed the experimental group at post-test on the task switching task, showing that this group improved in their trained domain (for similar findings see also [47]).

A tentative explanation is that divergent thinking generally decreases over time without training. However, this contrasts behavioral results of prior divergent thinking training studies: effectiveness of a broad range of divergent thinking training programs has been established in a broad range of populations (e.g., children, adolescents, adults), including training programs similar in length and content to the current AUT-training (see meta-analysis [48]). A potential explanation for the lack of a training effect in the expected direction may lie in the invariant repetitive design without external guidance and motivation. Although motivation scores did not change during the training, they were average. It has previously been argued that training programs with less active guidance are negatively related to the training effect size [48]. Providing active guidance during the training, including information about the creativity construct and the goal of the training may therefore be an essential component of a successful divergent thinking training. Hence, including active guidance and an incentive to maintain high motivation are recommended in future studies.

We also tested the effects of divergent thinking training on a near transfer task (AUT-brick) and a far transfer task (CAT), but the results showed that performance did not differ between the experimental and control group at post-test. This is not uncommon in training studies [49], [50]. That is to say, even though performance on trained tasks is generally well established, transfer to other domains is not consistently found [49]. One possibility is that the current task should be seen as a ‘practice’ task rather than a ‘training’ task where task difficulty is adapted based on performance. It was previously argued that transfer is only found when task difficulty increases over training [19], [47].

### Connectivity patterns in relation to training

The final question that we addressed was whether connectivity between brain areas implicated in divergent thinking is dependent on experience and may therefore be modified by training. Contrary to our predictions, the divergent thinking training as compared to the rule-switching (LGT) training was not significantly related to changes in seed-based or whole-brain RS functional connectivity over time. Thus, in comparison to the control group, we did not observe a significant change in seed-based and whole-brain RS functional connectivity patterns after the 8-session AUT-training. To the best of our knowledge, this is the first study to investigate the relationship between RS connectivity and a divergent thinking training. However, prior studies using different cognitive training programs did report RS connectivity changes in adults after 5–6 weeks working memory training [18] or 3 months reasoning training [20]. A limitation of the current study is that the groups were relatively small, however, training effects on brain activity have previously been observed using similar sample sizes [51]. Alternatively, it is possible that training effects on RS connectivity are observed in adults but not in adolescents (see also [18]), yet this interpretation is unlikely given the high level of brain plasticity associated with adolescent brain development [15]. Future studies should include participant groups of different ages to test this hypothesis.

When we tested whether behavioral improvement could be predicted by pre-test RS functional connectivity, we found that changes in AUT-scanner fluency were negatively related to connectivity between the left SMG and occipital cortex, suggesting that an anti-correlation between the SMG and occipital cortex may be a predictor of the trainability of divergent thinking. That is to say, we found that a weaker anti-correlation between SMG and occipital cortex was associated with less performance improvement. Anti-correlations between brain areas, also referred to as functional segregation, have previously been associated with better cognitive performance [52], [53]. While not predicted in advance, this finding may indicate that a stronger functional segregation between the SMG and occipital cortex may benefit the possibility to think of more alternative ways to use objects for a similar interpretation of stronger connectivity predicting negative training outcome.

### Conclusions and considerations for future studies

Taken together, the current study showed that divergent thinking performance can be linked to connectivity strength in a task-relevant network in adolescents. Prior studies investigating the neural mechanisms underlying divergent thinking generally show inconsistent findings that are, at least in part, attributed to differences in the operationalization of divergent thinking [4], [5], [54]. However, using a nearly similar version of the AUT-scanner, the role of the MTG (including the AG) and the SMG during the AUT has now been established in four separate studies [8]–[10]. We showed that the MTG and the SMG have connectivity patterns which are predictive for divergent thinking performance and changes in divergent thinking performance. This study should be followed up by studies with larger sample sizes, different age selections, and more diverse training programs. Nevertheless, the current study provides the first evidence for the relation between RS functional connectivity and divergent thinking in adolescence and thereby takes an important step in unraveling the relationship between creative cognition and functional brain connectivity.
